# Changes in concentrations of melatonin, PlGF, and cytokines in women with preeclampsia

**DOI:** 10.25122/jml-2022-0283

**Published:** 2023-03

**Authors:** Ruslan Florovych Savka, Andrii Mykolaiovych Berbets, Adrian Mykhailovych Barbe, Oleksandr Mykhailovych Yuzko, Mihaela Raluca Radu

**Affiliations:** 1 Department of Obstetrics and Gynecology, Bukovinian State Medical University, Chernivtsi, Ukraine; 2 Materno-Fetal Assistance Excellence Unit, Polizu Clinical Hospital, Alessandrescu-Rusescu National Institute for Mother and Child Health, Bucharest, Romania

**Keywords:** pregnancy, preeclampsia, melatonin, placental growth factor, cytokines, CNS – central nervous system, FLT-1 – Fms-related tyrosine kinase-1, IL – interleukin, PE – preeclampsia, PlGF – placental growth factor

## Abstract

Preeclampsia (PE) is a pregnancy-related disorder that significantly increases the risk of maternal and fetal morbidity and mortality. Melatonin, a potent antioxidant, has been suggested to mitigate oxidative stress and associated damage in various pathological conditions. Placental growth factor (PlGF) plays a vital role in placental development by promoting angiogenesis. This study aimed to investigate whether the levels of melatonin, cytokines, and PlGF were higher in the venous blood of women with preeclampsia during the third trimester of pregnancy compared to those with uncomplicated pregnancies. The study involved 32 women with preeclampsia and 33 healthy pregnant women as a control group. The concentrations of melatonin and PlGF were significantly lower in women with preeclampsia compared to healthy pregnant women. Specifically, the mean level of melatonin in the preeclampsia group was 30.98 pg/ml and 55.20 pg/ml in the control group (p=0.029). Similarly, the mean level of PlGF in the preeclampsia group was 40.03 pg/ml and 213.31 pg/ml in the control group (p<0.0001). This suggests that alterations in the placental production of melatonin and PlGF may contribute to the development of preeclampsia. In contrast, we observed higher levels of the pro-inflammatory cytokine interleukin-6 (IL-6) and the anti-inflammatory cytokine interleukin-10 (IL-10) in the preeclampsia group than in the control group. The mean concentration of IL-6 in the PE group was 270.79 pg/ml, whereas the control group had 224.30 pg/ml (p=0.022). Similarly, the mean concentration of IL-10 in the PE group was 41.90 pg/ml and 30.73 pg/ml in the control group (p=0.018). In women with uncomplicated pregnancies, the interaction between pro-inflammatory interleukine-6 and melatonin can be described by equality of statistical regression.

## INTRODUCTION

Preeclampsia (PE) is a human pregnancy-specific systemic disorder of the mother-placenta-fetus system [1]. The condition is characterized by the onset of high blood pressure after 20 weeks of gestation, as well as damage to other organs, primarily kidneys, liver function disorders, changes in blood status, and often fetal growth restriction [2]. Thus, the key link in the pathogenesis of preeclampsia is placental dysfunction. In addition to the production of pathological molecules by the placenta, in particular, sFlt-1, which is a consequence of poor trophoblast invasion in the first trimester of pregnancy [3], a significant role in the development of the mentioned pathological condition is played by hypoxic-reperfusion damage to the placental tissue [1]. This damage is directly related to the excessive secretion of anti-angiogenic molecules by the placental tissue [2] and insufficient pro-angiogenic ones, in particular, the placental vascular growth factor, PlGF [4]. Thus, a massive endothelial dysfunction occurs in the maternal body, which progressively leads to a growth in the resistance of peripheral vessels and the activation of procoagulant mechanisms and the immune response [2], in particular, to an increase in the production of pro-inflammatory and a decrease in anti-inflammatory cytokines [5,6]. This highlights the potential risks associated with the progression of preeclampsia, which can lead to a pathological condition characterized by high blood pressure, multiorgan failure, and in severe cases, cerebral edema and seizures [1]. Even in highly developed countries, preeclampsia is the cause of up to 15% of cases in maternal mortality statistics [2]. After childbirth, pathological changes in the mother's body caused by preeclampsia do not regress immediately [1]. Women who have experienced this complication of pregnancy are at higher risk of developing hypertensive disease in the future. This is due to the residual phenomena of endothelial dysfunction, which, in turn, is the result of oxidative stress of endothelial cells [2]. Moreover, some researchers believe that children born to mothers with preeclampsia may also be at higher risk for health problems later in life. Such children are more likely to develop hypertension, as well as metabolic disorders such as type 1 diabetes and obesity [7].

Therefore, although preeclampsia is considered a disease of the mother, it should rather be considered a pathological state of the placenta, which affects the health of both the mother and the fetus. Since oxidative stress is a key factor in placental disorders [8], many researchers consider melatonin a potentially useful option for treating preeclampsia [1, 9]. As already mentioned, melatonin is a powerful natural antioxidant. In this case, it acts directly and indirectly. The direct path consists of the effective removal of atomic oxygen species from cells directly by the melatonin molecule, while the indirect path includes the activation of endogenous antioxidant enzymes, namely: glutathione peroxidase, glutathione reductase, superoxide dismutase, and catalase [1]. During a normal pregnancy, the level of melatonin progressively increases [10, 11]; there are data on the joint expression of melatonin and oxytocin receptors in the myometrium before and during childbirth [12]. There are reports of a decline in melatonin levels in preeclampsia when the degree of reduction correlates with the severity of the process [13] and of a decrease in the expression of melatonin receptors in placental tissue in the case of fetal growth restriction [14].

As mentioned above, one of the main pathophysiological aspects of preeclampsia is the production of the molecule sFlt-1 (soluble FMS-like tyrosin-kinase-1) by the placenta [15]. The excessive presence of this molecule, among other anti-angiogenic factors, in the blood plasma of pregnant women is associated with arterial hypertension, proteinuria, thrombocytopenia, glomerular endotheliosis, increased liver enzymes, etc. [15–18]. In a physiological state, this molecule is located on the surface of endotheliocyte membranes and is a receptor for pro-angiogenic agents, in particular, for the placental growth factor PlGF [18]. Hypoxia causes post-translational cleavage of this membrane receptor endoglin into its soluble form [15]. As a result, sFLT-1 exerts an anti-angiogenic effect and worsens the condition of the endothelium, while the production of placental growth factor PlGF decreases in these conditions. Dysfunction of the endothelium leads to an increase in its permeability, which results in tissue swelling [15]. In addition, the oxidative stress of the placental tissue causes an increase in the level of activin, which is also an anti-angiogenic factor and enhances vasoconstriction by stimulating the production of endothelin in the endothelium of vessels [19], which, in combination with an increase in the concentration of sFlt-1, leads to the rapid progression of preeclampsia.

In pregnant women with preeclampsia, a decrease in night-time concentrations of melatonin is noted, compared to healthy pregnant women [11], which may indicate a disorder in the functioning of the pineal gland in preeclampsia. However, the decrease in melatonin levels in this pathology is caused not only by changes in the work of the pineal gland: it is known that the expression of the two most important enzymes necessary for the synthesis of melatonin, namely, alkylamine-N-acetyltransferase and hydroxy indole-O-methyltransferase, is significantly reduced in placental tissue in women diagnosed with preeclampsia, as well as the expression of melatonin receptors of both types (M1 and M2). Therefore, insufficient melatonin synthesis by the placenta in preeclampsia has been accurately confirmed [20].

Regarding changes in the cytokine profile in preeclampsia, there is evidence that the levels of pro-inflammatory cytokines, namely TNF-α and IL-6, are increased in preeclampsia, while the concentrations of anti-inflammatory cytokines, namely IL-4 and IL-10, are decreased [21]. Melatonin, on the contrary, reduces the secretion of pro-inflammatory cytokines, in particular, TNF-α [22], and increases the production of anti-inflammatory cytokines, namely, IL-10 [23]. Therefore, the normalization of melatonin levels can be a promising direction in the treatment of preeclampsia in pregnant women.

There is very little information in the contemporary literature on the relationship between melatonin levels and the placental growth factor PlGF, which is important in the pathogenesis of preeclampsia development. However, a recent study found that the levels of melatonin and placental growth factor decrease in umbilical cord blood collected during childbirth in women whose pregnancy was complicated by fetal growth restriction [24].

Therefore, it can be concluded that preeclampsia is a complex pathological process, where a significant role contributing to its development is the decrease of melatonin levels in the pregnant woman's body, which is a consequence of the dysfunction of the pineal gland and the placenta [25]. The purpose of our study was to uncover certain aspects of this pathophysiological mechanism.

## MATERIAL AND METHODS

### Study design

We conducted an analytical case-control study to investigate changes in the levels of melatonin, PlGF, IL-6, and IL-10 cytokines in women with preeclampsia compared to women with uncomplicated pregnancies. Finally, we aimed to establish if there was a correlation between PlGF and melatonin concentration and the development of preeclampsia.

### Subjects

The study was conducted at the Department of Pregnancy Pathology of the Chernivtsi Regional Perinatal Center. The study group included 32 women with confirmed preeclampsia, diagnosed by systolic blood pressure of ≥160 mmHg or diastolic blood pressure of ≥110 mmHg and proteinuria of ≥300 mg per 24-hour urine collection. All women in the study group had pregnancy term within 30-32 weeks of pregnancy, confirmed by due date calculation based on the first pregnancy trimester ultrasound examination (11-13 weeks).

The control group included 33 women with uncomplicated pregnancies who received care at the Women's Consultation Department at the same facility.

Patients with clinically significant extragenital diseases (chronic hypertension, obesity, pre-gestational and gestational diabetes, endocrine disorders, anemias, etc.) were excluded from the study and control groups.

The study group had a mean age of 28.59 years (95% confidence interval for mean 27.3 – 29.88 years), while the control group had a mean age of 31.24 years (28.97-33.51). Women in the study group weighed an average of 74.03 kg (71.72-76.33 kg), whereas those in the control group had an average weight of 65.63 kg (62.66-68.60 kg). Both groups delivered their babies vaginally after completing 37 weeks of pregnancy. The mean delivery term was 38.09 weeks in the study group and 38.67 weeks in the control group. At the time of the blood sampling, there were no significant differences between the groups in terms of age, ethnicity, body weight, or pregnancy term. All patients delivered their children vaginally upon completing at least 37 weeks of pregnancy. The study excluded cases of severe fetal distress that required a cesarean section, obstetrical forceps, or vacuum extraction of the fetus.

### Biochemical analyses

Melatonin and PlGF concentrations were measured using an ELISA kit manufactured by "IBL" (Germany), and pro-inflammatory (IL-6) and anti-inflammatory (IL-10) cytokines were measured using diagnostic kits manufactured by "Vector Best" (Ukraine). Venous blood samples were collected by vein puncture at 9:00 a.m. from fasting patients, and all analyses were performed at the same time.

### Statistical analyses

Statistical data was calculated using MedCalc software, developed by "MedCalc Software," based in Ostend, Belgium. The Mann-Whitney U-test was used to evaluate results for small groups, and a P-value of 0.05 was considered significant. Correlation and regression statistical analyses were conducted using the same software. We did not find any statistical difference in the daytime of delivery between groups.

## RESULTS

**Figure 1:**
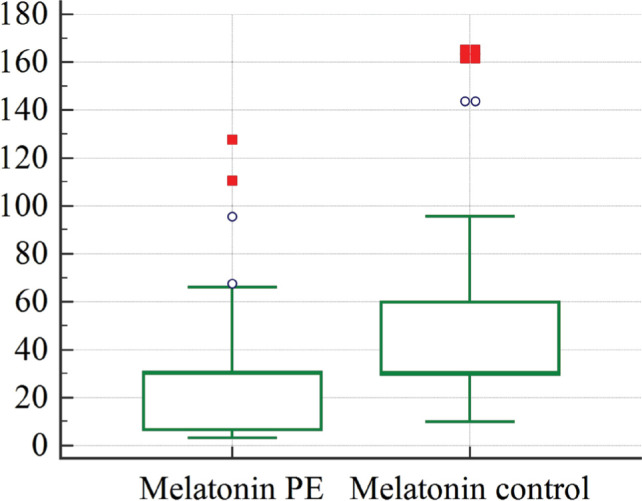
Graphical comparison of melatonin concentrations in women diagnosed with preeclampsia: study group (Melatonin PE), control group (Melatonin control).

Table 1 presents the melatonin levels in the venous blood samples obtained from the patients in the studied groups. The level of melatonin in the venous blood of women with diagnosed preeclampsia was significantly lower (P=0.029) compared to healthy women (Figure 1). As a result, we can conclude that melatonin levels in maternal venous blood are reduced during pregnancy in cases of preeclampsia.

**Table 1:** Melatonin levels in venous blood obtained during labor from women diagnosed with PE.

	Women with preeclampsia (n=32)	Control group (n=33)	P-value
**Melatonin (pg/ml)**	30.98 (19.78–42.17)	55.20 (36.23–74.17)	**0.029**

The P-value was calculated using the Welch test (assuming unequal variances). A 95% confidence interval for the mean is indicated in brackets.

**Figure 2:**
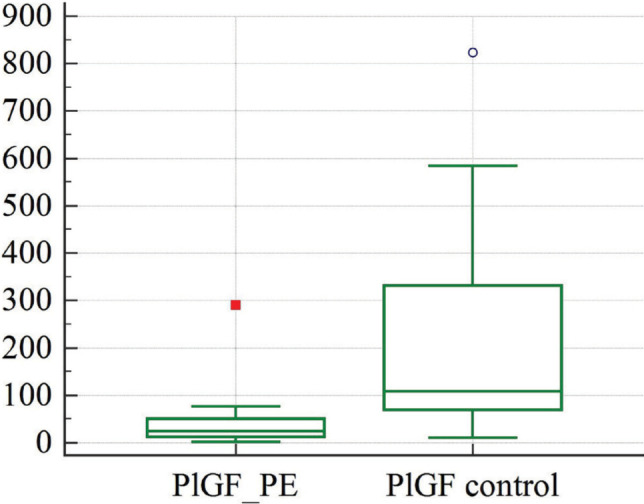
Graphical comparison of PlGF concentrations in women diagnosed with preeclampsia: study group (PlGF_PE), control group (PlGF control).

Table 2 shows the levels of PlGF in venous blood taken from patients in both groups. The concentration of PlGF in venous blood taken from women with diagnosed preeclampsia was 5 times lower than in women with uncomplicated pregnancies (p<0.0001) (Figure 2).

**Table 2:** PlGF levels in umbilical blood taken during labor from women diagnosed with PE.

	Women with preeclampsia (n=32)	Control group (n=33)	P-value
**PlGF (pg/ml)**	40.03* (20.39–59.67)	213.31 (144.76–281.85)	**<0.0001**

The P-value was calculated using the Welch test (assuming unequal variances). A 95% confidence interval for the mean is indicated in brackets.

**Figure 3:**
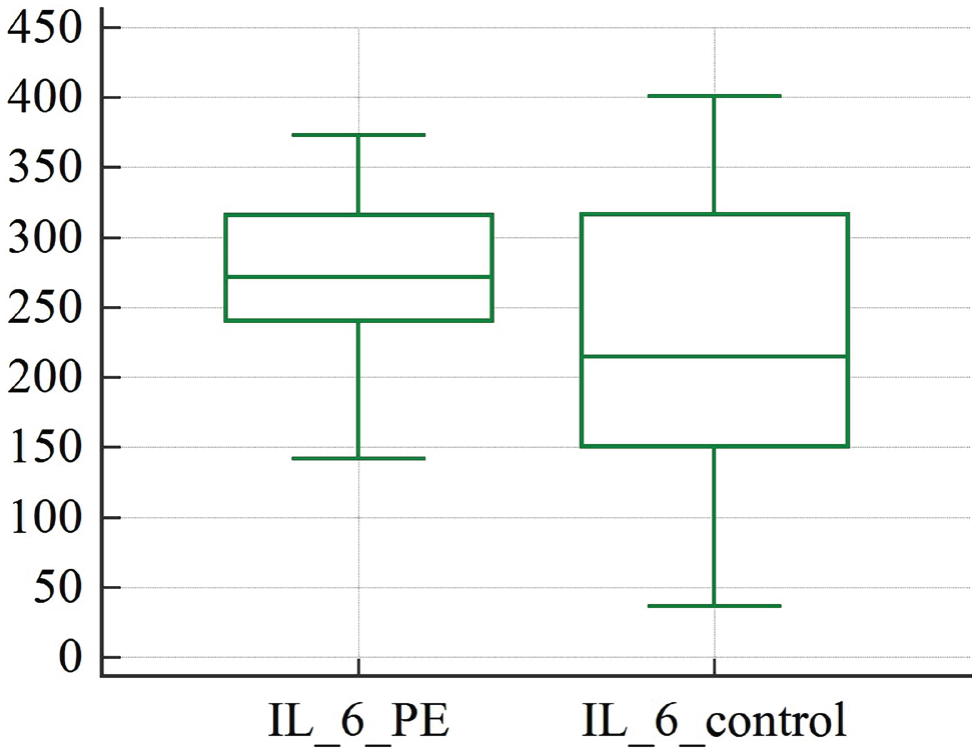
Graphical comparison of pro-inflammatory IL-6 concentrations in women diagnosed with preeclampsia: study group (IL_6_PE), control group (IL_6_ control).

**Figure 4:**
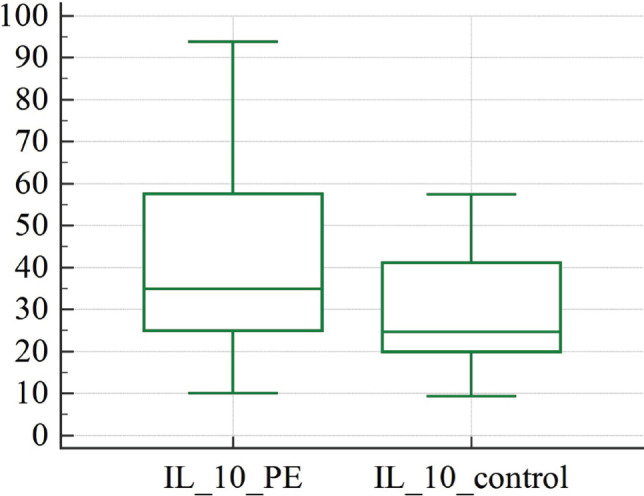
Graphical comparison of anti-inflammatory IL-10 concentrations in women diagnosed with preeclampsia: study group (IL_10_PE), control group (IL_10_ control).

Table 3 shows the levels of the pro-inflammatory cytokine IL-6 and the anti-inflammatory cytokine IL-10 in the venous blood of the patients investigated. There was a significant difference in cytokine concentrations between the studied groups. We discovered an increase in both IL-6 and IL-10 concentrations in the PE group compared to patients with uncomplicated pregnancies (Figures 3 and 4).

**Table 3:** IL-6 levels in the umbilical blood of women diagnosed with PE.

	Women with preeclampsia (n=32)	Control group (n=33)	P-value
**IL-6 (pg/ml)**	270.79 (248.26–293.31)	224.30 (190.94–257.67)	**0.022**
**IL-10 (pg/ml)**	41.90 (33.88–49.93)	30.73 (25.97–35.49)	**0.018**

The P-value was calculated using the Welch test (assuming unequal variances). A 95% confidence interval for the mean is indicated in brackets.

**Figure 5:**
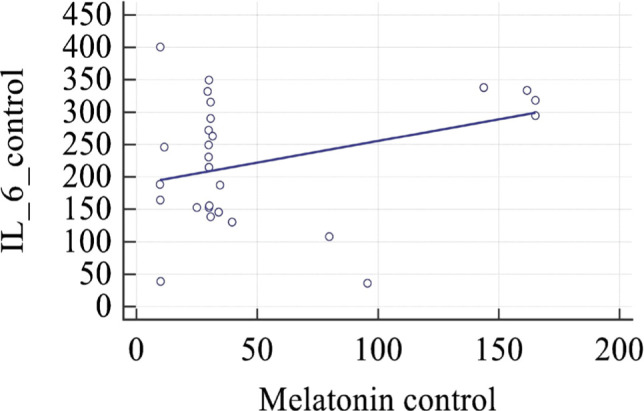
Scatter diagram and regression line of concentrations of pro-inflammatory IL-6 and melatonin in the control group.

We did not find any significant correlation between the studied indexes. Nevertheless, we calculated the regression equalities, including assayed biochemical agents in the study and control groups. It was established that in the group of healthy pregnant women, the regression equality that includes the levels of interleukin-6 and melatonin could be described as:

y=188.4401+0.6700x (P=0.0373)

where: y – is the level of interleukin-6 in the blood of a pregnant woman in the control group; x – is the level of melatonin in the blood of a pregnant woman in the control group.

Parameters of the regression: intercept coefficient = 188.401 (standard error 23.2748, P<0.0001), slope coefficient = 0.6700 (standard error = 0.305, P<0.0373).

The scatter diagram with the regression line is depicted in Figure 5.

## Discussion

We determined that pregnant women with preeclampsia (PE) had significantly lower melatonin levels in their venous blood during the third trimester of pregnancy compared to patients with uncomplicated pregnancies (Figure 1). This finding is consistent with previous studies [11, 20].

The observed melatonin deficiency may exacerbate oxidative stress in preeclampsia, leading to increased levels of circulating reactive oxygen and reactive nitrogen species, which play important roles as secondary messengers in various intracellular signaling pathways. However, these species can also have critical effects on pathological processes affecting pregnant women [26]. The induction of neutrophil adhesion to the endothelium, accompanied by the release of cytokines and the activation of inflammatory response-associated signaling pathways, is one of the most important signaling pathways activated by reactive oxygen and nitrogen species [26–28]. In addition, poor trophoblast invasion, which is confirmed as one of the main pathological aspects of preeclampsia [29], results in placental hypoxia, accelerating the apoptotic cascade in villous trophoblast. In the case of abnormal trophoblast invasion, the production of several other factors, such as leukocyte and platelet membrane particles, cytokines, growth factors, and angiogenic factors, is also altered, and these factors will then interact with maternal vascular endothelium, which may already be damaged [29]. This view, as we consider it, is mainly confirmed by our study.

Furthermore, our research revealed that PlGF concentrations in venous blood were decreased in cases where the pregnancy was complicated with PE (Figure 2). This finding is not unexpected and can be considered one of the biochemical markers of preeclampsia in pregnant women of the study group. Previous studies have reported changes in PlGF concentrations in maternal blood, predominantly in cases of preeclampsia [30]. PlGF is known to be responsible for angiogenesis in the placenta and actively binds to VEGFR-1 (vascular endothelial growth factor-1 receptor-1) and its soluble variant sFLT-1 (soluble fms-like tyrosine kinase-1), which is the key molecule in the pathogenesis of preeclampsia, primarily due to endothelial damage [31].

Our study uncovered significant changes in cytokine levels between women with preeclampsia and those with uncomplicated pregnancies. Notably, we discovered that pro-inflammatory IL-6 and anti-inflammatory IL-10 levels were higher in women with preeclampsia compared to women with uncomplicated pregnancies (Figures 3 and 4). This increase in pro-inflammatory cytokines, particularly IL-6, is a well-established feature of preeclampsia, as evidenced by a recent study demonstrating elevated levels of IL-6 and its receptor gp130 in endothelial cells from preeclampsia patients [32]. Conversely, most studies have reported decreased levels of anti-inflammatory cytokine IL-10 in preeclampsia cases.

For illustration, a study of a preeclampsia model in rats with decreased uterine perfusion found that placental ischemia was associated with lower levels of IL-10 and endothelial cell dysfunction [33]. As a significant immunosuppressive cytokine, IL-10 is mainly released by Th2 cells, macrophages, natural killer cells, granulocytes, dendritic cells, and B cells triggered by autoantigens. Normal pregnancy is characterized by a shift towards Th2 immunity relative to Th1 immunity, which is changed in cases of preeclampsia [34]. Inhibiting IL-10 during the second half of pregnancy in mice reduced fetal growth but had no effect on the gestational duration or fetal outcome, demonstrating the important but non-essential role of IL-10 in fetal growth [35, 36]. Individual levels of IL-10 in preeclampsia and normotensive patients were not significantly different in certain human research [35, 36]. In addition to the elevated levels of pro-inflammatory cytokine IL-6, our study also found elevated levels of anti-inflammatory cytokine IL-10 in women with preeclampsia (Figure 4). We hypothesize that the inclusion of only women with preeclampsia confirmed by proteinuria and hypertension, as well as the exclusion of patients with fetal growth restriction and fetal distress from the study group, may have contributed to the elevated levels of IL-10. This suggests that IL-10 may play a protective role for the fetus in cases of preeclampsia. In women with uncomplicated pregnancies, we calculated the equality of regression, which had statistical significance, and described the interaction between pro-inflammatory interleukin 6 and melatonin.

On the other hand, we did not find such statistical significance in the study group, probably due to altered signaling pathways in the case of preeclampsia. Relying on the abovementioned, we propose that melatonin acts as a modulator of the inflammatory response of placental tissue. Melatonin's anti-inflammatory and anti-oxidative properties are well acknowledged, and they simply cannot be emphasized enough in the case of PE.

Our study has several strengths, including strict inclusion criteria (the diagnosis of PE was confirmed in all cases included in the study group), exclusion of cases with compromised fetal condition from the study group, venous blood sampling at the third pregnancy trimester on a fixed daytime, and estimation of regression links between studied indexes. However, a limitation of our study is that the sample size was relatively small due to ethical constraints.

## Conclusion

Melatonin and PlGF concentrations in venous blood are significantly lower in women whose pregnancies were affected by preeclampsia in the third pregnancy trimester compared to uncomplicated pregnancy cases. In contrast, pro-inflammatory IL-6 and anti-inflammatory IL-10 levels were higher in preeclampsia patients compared to normal pregnancies. We suggest that the placental dysfunction mainly results in the damage of the synthesis of antioxidant melatonin and pro-angiogenic PLGF, which causes impairment of the maternal, placental, and fetal tissues and consequently provokes clinical manifestation of preeclampsia. The subclinical inflammatory process also contributes to the deterioration of placental health, as evidenced by the increased levels of IL-6 detected in patients diagnosed with preeclampsia. In our study, we observed an increase in IL-10 levels in the study group, which we believe to be a compensatory reaction to prevent premature birth. In women with uncomplicated pregnancies, the interaction between pro-inflammatory IL-6 and melatonin can be described by equality of statistical regression.
